# Anticoagulation Use prior to Common Dental Procedures: A Systematic Review

**DOI:** 10.1155/2019/9308631

**Published:** 2019-06-02

**Authors:** Johnny Chahine, Marwan N. Khoudary, Samer Nasr

**Affiliations:** ^1^Department of Internal Medicine, Cleveland Clinic Foundation Fairview Hospital, Cleveland, OH, USA; ^2^Department of Conventional and Surgical Endodontics, Senior Instructor, St Joseph University, Beirut, Lebanon; ^3^Head of Department of Electrophysiology and Cardiology, Mount Lebanon Hospital, Lebanese University, Beirut, Lebanon

## Abstract

Currently, the number of patients on oral anticoagulation is increasing. There is a paucity of data regarding maintaining oral anticoagulation (especially novel oral anticoagulants) around the time of specific dental procedures. A dentist has three options: either to stop anticoagulation, to continue it, or to bridge with heparin. A systematic review of 10 clinical trials was conducted to address this issue. It was found that continuing anticoagulation during dental procedures did not increase the risk of bleeding in most trials. Although none of the studies reported a thromboembolic event after interruption of anticoagulation, the follow-up periods were short and inconsistent, and the heightened thromboembolic risk when stopping anticoagulation is well known in the literature. Heparin bridging was associated with an increased bleeding incidence. We recommend maintaining oral anticoagulation with vitamin K antagonists and novel oral anticoagulants for the vast majority of dental procedures along with the use of local hemostatic agents.

## 1. Introduction

The use of anticoagulation is increasing in the population, and it is almost a daily occurrence to have a patient presenting for a dental procedure on vitamin K antagonists (VKAs) or novel oral anticoagulants (NOACs). Before considering stopping oral anticoagulation periprocedurally, the physician must balance between the risk of thromboembolism and bleeding associated with that procedure [1].

In the case of a surgical procedure, three possibilities are available: first to maintain warfarin, second to interrupt it, and third to withhold it and to do heparin bridging before the procedure. Stopping warfarin before a procedure can be detrimental to the patient's health, increasing thromboembolism and mortality rates [2, 3]. Thromboembolic events were seen in 0.7% to 1.1% in patients who stopped anticoagulation before an invasive procedure [1, 4]. A survey showed that most German dentists tend to stop VKAs before dental procedures [5]. Also, dentists registered in Michigan had nonuniform approaches towards patients on warfarin [6].

Concerning NOACs, a four-year cross-sectional study showed no significant bleeding when continuing anticoagulation with dental procedures, regardless of the invasiveness of the procedure [7]. The analysis of the RE-LY trial revealed that no significant differences in bleeding and thromboembolic complications exist between dabigatran and warfarin [8]. Although dabigatran has no antidote, it has a short half-life. Thus, a quick reversal of anticoagulation is possible if needed [8]. In an analysis of the EINSTEIN studies, rivaroxaban, another NOAC, has caused less major hemorrhagic events than AVK/bridging therapy when treating deep venous thrombosis and pulmonary embolism [9].

The American College of Chest Physicians Evidence-Based Clinical Practice Guidelines 9th edition recommends “either to maintain VKAs along with an oral prohemostatic agent or to interrupt them a couple of days before minor dental procedures.” A need for bridging was not mentioned [10]. The European Society of Cardiology in 2009 [11], along with the American Academy of Oral Medicine in 2016 [12], recommends, for the majority of outpatient dental procedures, continuing VKAs if the international normalized ratio (INR) is in the therapeutic range. Because there is not enough data available regarding NOACs, the American Dental Association suggests continuing anticoagulation for the vast majority of dental procedures unless the patient is at a very high risk of bleeding, when a physician referral might be appropriate before the procedure [13].

While maintaining anticoagulation with VKAs during dental interventions, the postoperative bleeding risk might be reduced by adopting local hemostatic measures. Many agents were found to be effective: tranexamic acid mouthwash [14, 15] for 2 days [14], oxidized cellulose and sutures [16], gelatin sponge [17, 18], fibrin adhesives [19], HemCon Dental Dressing [20–22], platelet-rich plasma gel [23], and Histoacryl glue [24]. However, some obstacles exist that limit the use of those agents, for example, the high cost of fibrin glue [15, 16] and the complex technique of tranexamic acid usage [25]. On the other hand, a Serbian study showed that local pressure is sufficient for adequate hemostasis in most cases of teeth extraction if INR is less than or equal to 3 [26]. It is noteworthy that suturing is not always necessary and should be reserved for instances where local hemostasis fails or when there is extensive tissue damage [17].

Although the data on VKAs are quite extensive and knowing that the bleeding risk in patients on NOACs might be higher, we are attempting a review of the literature of both VKAs and NOACs in the setting of a dental procedure. Rather than dividing the dental procedures largely into mild, moderate, and high risk of bleeding, we will attempt the evaluation of the risk of bleeding periprocedurally with specific dental procedures.

## 2. Methods

We have performed a systematic review of the literature on PubMed regarding anticoagulation during dental procedures. The keywords used were as follows: anticoagulation, anti-coagulation, Vitamin K, bridging, dental, dentist, tooth, teeth, and oral. The range of the studies is from 1996 to 2016, with most of the studies being after 2000.

From each study, we collected the following data: the number of patients, age, indications for anticoagulant treatment, exclusion criteria, the regimen of anticoagulation, bridging used, the procedure done, local hemostatic agents used, preoperative INR, target INR before undergoing the procedure, thromboembolic outcome, maximum follow-up period, and bleeding characteristics.

Concerning the latter, every study had a unique tool to assess and quantify bleed. We reported the outcomes accordingly.

This review aims to suggest recommendations for every specific dental procedure when it comes to continuing or interrupting VKAs and NOACs.

For every procedure, we determined the risk of bleeding and the recommendations regarding VKAs and NOACs. We attempted to base our recommendations on the results of well-established randomized controlled trials (RCTs) and controlled clinical trials (CCTs). When data are lacking, we reported an expert's opinion. The dental procedures assessed were as follows: surgical teeth extraction, implant surgery, excision of cystic formations, biopsies, alveoloplasty, frenectomy, periodontal surgeries, and microsurgical endodontics (apicectomy).

## 3. Results

### 3.1. Study Selection

**Figure 1:**
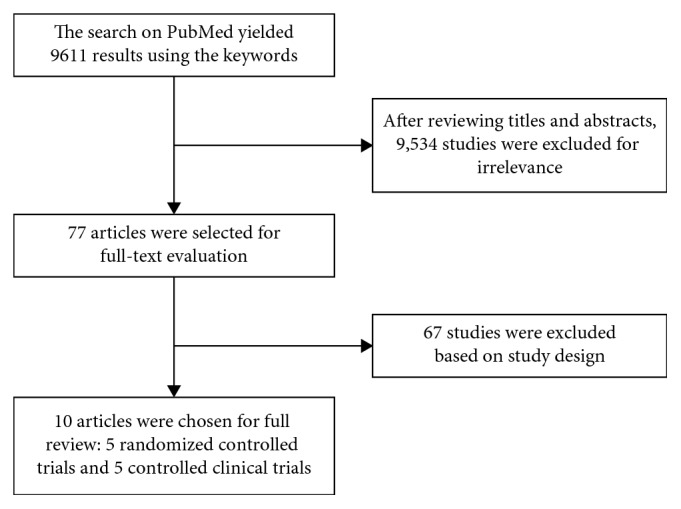
Process of selection of the studies.

The process of selection of the studies is summarized in Figure 1. Ten trials were selected: 5 RCTs [17, 27–30] and 5 CCTs [31–35]. The studies date from 1996 till 2016.

### 3.2. Participant Characteristics

The total number of participants was 1331; at least 457 of them had their anticoagulation uninterrupted during the procedure. Most studies consisted of two groups: the first had oral anticoagulation continued during the dental procedure, the other had it stopped a few days before, with or without bridging with heparin. Warfarin was the main oral anticoagulant used, although some studies had other VKAs and only one studied NOACs. The bulk of the studies practiced local hemostatic measures after the surgeries. The primary procedure studied throughout was dental extractions, with or without a raise of a mucoperiosteal flap. The indications for anticoagulant treatment were multiple, and the follow-up period extended from 1 day to 1 month. Most studies had their target INR within the therapeutic range in the anticoagulant group and therefore their preoperative INR falling within that range. Patients at risk of bleeding were predominantly excluded, like those with liver disease, renal disease, and coagulation abnormalities and those on drugs that increase that risk (Table 1).

### 3.3. Study Outcomes

Every study had its protocol to assess bleeding outcome. A statistically significant difference in bleeding among groups was only observed in 2 studies: the first showing increased bleeding when bridging with LMWH [32] the second showing an increase in mild bleeding in VKA group when compared with no anticoagulation [33]. Only 4 patients across the 10 studies were reported to need hospitalization due to bleeding. The number of teeth extracted was associated with an increased risk of bleeding in one study [32]. This relationship was not seen in two other trials [27, 29]. There was no association between INR levels and postoperative bleeding [17]. A thromboembolic event was not observed in any of the studies, even in patients who interrupted their anticoagulation.

All studies recommended oral anticoagulation to be continued if INR is in the therapeutic range or inferior to 3. When maintaining oral anticoagulation, some studies found local hemostasis helpful. Bridging with LMWH [32] or giving heparin with reduced VKA dose [30] was found to increase the risk of bleeding (Table 2).

### 3.4. Recommendations

Most evidence exists for surgical teeth extraction (5 RCTs and 4 CCTs). Concerning the rest of the procedures, the studies are mostly CCTs. For periodontal surgeries and endodontic microsurgeries, no controlled trials are available yet. After being certain that the patient is not overly anticoagulated and the drugs are adjusted based on creatinine level, we do recommend continuing anticoagulation in the vast majority of patients along with the use of local hemostatic agents. Although thromboembolic events were not seen in the trials studied (probably due to the short follow-up periods), it is well established that interrupting anticoagulation increases thromboembolic risk; therefore, this should be avoided as much as possible (Table 3).

## 4. Discussion

Bleeding during dental procedures occurs mostly in patients that are overly anticoagulated. A simple procedure can turn into a nightmare if the patient is on an AVK and his INR is above 4, or if he is on a NOAC with renal dysfunction.

When an anticoagulated patient presents for a dental procedure, the dentist has three main options: to continue the same dose of oral anticoagulation with local hemostatic agents, to diminish the dose, or interrupt it altogether a few days before [33]. Our systematic review has revealed that the first option is the best in most procedures, with none of the 10 studies recommending the remaining two options since no statistically significant difference in postoperative bleeding existed between most groups continuing and interrupting oral anticoagulation. Other studies have also come to the same conclusion: if INR is reasonable and local hemostatic measures adopted, there is no adverse outcome for continuing oral anticoagulation in dental procedures [38–42]. We recommend that VKAs must be continued in all surgical procedures if INR is in the therapeutic range. As for NOACs, they must also be maintained in most procedures. Local hemostatic agents are mostly needed in both cases.

LMWH bridging has been deemed not necessary in dental procedures [17], or even harmful by increasing bleeding risk [32, 43] without altering the INR level. It has been found that heparin and reduced acenocoumarol [30] increase bleeding risk as well after dental procedures. Also, trying to replace heparin bridging with oral vitamin K one day before the procedure was unsuccessful as vitamin K did not adequately correct INR [44].

Special measures were taken in most studies to diminish bleeding risk [17, 27, 28, 33–35], like reducing soft tissue and bone injuries and minimizing the need to raise a mucoperiosteal flap during the procedures. However, it must be noted that whether a mucoperiosteal flap raise was needed or not in dental extractions [17, 27, 28, 32, 34, 35], the outcome remained in favor of maintaining oral anticoagulation. Also, in implant surgery, bleeding risk was not associated with the invasiveness of the surgery [33].

There was no association between the number of teeth extracted and postoperative bleeding [27, 29, 30], except in one study [32]. In this particular study, the sample was relatively small, and the patients were their own control, unlike the other studies. As a matter of fact, bleeding mainly occurs where local inflammation is severe [18].

In contrast to previous studies [2, 3] and in line with others [4, 25], a short interruption of oral anticoagulation did not seem to increase the risk of thromboembolic events in the 10 trials. However, the follow-up period, extending from one day to one month, was relatively small, and the thromboembolic risk could not be fully assessed based on the trials.

Studies were divided between the ones which recommend the use of local hemostatic agents [17, 28–30, 35] and the ones which consider it unnecessary [31, 34]. Many case-control [39, 42] and cross-sectional [38, 40, 41] studies also recommended their use. Suturing was not deemed essential to assuring hemostasis [17, 29], and has many downsides: it predisposes to thromboembolism [29], lengthens healing time [17, 29], and accumulates aliments [17].

In brief, there is an immense need for cooperation between physicians and dental surgeons [17, 45]. Although they both admit lacking full knowledge concerning oral anticoagulation in dental surgeries, dentists and physicians tend to mutually criticize [46]. Multiple measures are proposed for better cooperation, like having common classes in schools and establishing guidelines together. If a physician referral is necessary prior to a dental procedure, the dental surgeon should inform the physician that major bleeding is less likely in most procedures and that most guidelines recommend the continuation of anticoagulation, since physicians tend to overestimate the risk of bleeding.

This review has many limitations. The methods of assessing bleed were not uniform across the studies, which make an accurate comparison of bleeding outcome challenging. All the studies had VKAs as their oral anticoagulants except one CCT which included NOACs. Moreover, there is a lack of RCTs dealing with procedures other than teeth extraction, which creates a gap in the literature for the remaining procedures. Except for Erden et al. and Souto et al., the indications for anticoagulation were multiple and variable. There is a need for RCTs for specific patient populations, as patients with atrial fibrillation, for example, may be more predisposed to have a thromboembolic event [47].

## 5. Conclusion

For the vast majority of dental procedures, VKAs and NOACs must be maintained. Local hemostatic agents should be applied, and special attention should be given to INR level and renal function. Stopping and reinitiating oral anticoagulation can be troublesome for both the physician and the patient with an increased risk of thromboembolic events, and the best approach is multidisciplinary.

## Figures and Tables

**Table 1:** Participant characteristics.

Author and year of publication	Design	Number of participants	Age (mean (range) or mean ± SD or mean ± SD (range))	Indications for anticoagulant treatment	Exclusion criteria	Regimen of anticoagulation	Bridging used	Procedure	Local hemostatic agents used	Target INR before the procedure	Preoperative INR (mean (range) or mean ± SD)	Maximum follow-up period
Campbell et al., 2000 [31]	CCT	60	Not mentioned in the study	Not mentioned in the study	Not mentioned in the study	*Experimental (n* *=* *12):* warfarin continued *Control (n* *=* *13):* warfarin stopped 72 to 96 hours before the procedure *Baseline group (n* *=* *35):* no anticoagulation used	None	Dental extractions, quadrant alveoloplasty, frenectomy	Not mentioned in the study	Not mentioned in the study	*Experimental:* 2 (1.2–2.9) *Control:* 2 (1.1–3) *Baseline group:* not done	1 day

Evans et al., 2002 [27]	RCT	109	*Experimental:* 67 (36–92) *Control:* 66 (30–93)	Not mentioned in the study	INR > 4 on the day of operation; liver disease; coagulopathies	*Experimental (n* *=* *57):* warfarin continued *Control (n* *=* *52):* warfarin stopped 2 days before the procedure	None	Dental extractions and mucoperiosteal flap sometimes raised	Oxycellulose with sutures	*Experimental:* INR less than 4 *Control:* INR less than 2	*Experimental:* 2.5 (1.2–4.7) *Control:* 1.6 (1.2–2.3)	7 days

Erden et al., 2015 [32]	CCT	36	46.8 ± 11.4 (28–72)	Prosthetic valve	If flap elevation is required; chronic liver and renal disease; being on drugs other than warfarin that could affect the liver function or hemostasis; if the patient did not have two teeth to be extracted from the same dental extraction	*First dental extraction (group A):* warfarin continued *Second dental extraction (15 days after the first) of the same individuals (group B):* warfarin stopped 5 days before the procedure with LMWH bridging	LMWH in group B	Dental extractions (more than one tooth from the same dental groups) and no mucoperiosteal flap raised	Oxycellulose dressing and sutures	INR less than 4	*Group A:* 2.5 ± 0.3 *Group B:* 1.1 ± 0.1	10 days

Sacco et al., 2007 [28]	RCT	131	*Group A:* 64 (29–87) *Group B:* 61 (29–86)	Not mentioned in the study	Thrombocytopenia less than 100 10^9^/L; chronic liver and renal disease	*Group A (n* *=* *66):* warfarin or acenocoumarol stopped until INR between 1.5 and 2 preprocedurally *Group B (n* *=* *65):* OAT continued	None	Dental extractions, excision of cysts, implant surgery, and mucoperiosteal flap raised in all patients	*Group A:* sutures only *Group B:* sutures, gelatin, oxycellulose, tranexamic acid	*Group A:* INR between 1.5 and 2 *Group B:* INR between 2 and 4	*Group A:* 1.77 ± 0.26 *Group B:* 2.89 ± 0.42	7 days
Al-Mubarak et al., 2007 [29]	RCT	214	*Group 1:* 52.3 ± 14.3 *Group 2:* 51.7 ± 14.7 *Group 3:* 48.7 ± 13.1 *Group 4:* 53.1 ± 13.7	Not mentioned in the study	Patients with a history of chronic renal or liver disease and patients on drugs that could affect liver function or hemostasis, other than warfarin	*Group 1 (n* *=* *48):* no suturing and warfarin stopped 2 days before the procedure *Group 2 (n* *=* *58):* no suturing and warfarin continued *Group 3 (n* *=* *56):* suturing done and warfarin stopped 2 days prior to the procedure *Group 4 (n* *=* *52):* suturing done and warfarin continued	None	Dental extractions	Multiple agents used in all groups *Groups 3 and 4:* sutures	Not mentioned in the study	*Group 1:* 1.8 ± 0.4 *Group 2:* 2.4 ± 0.5 *Group 3:* 1.9 ± 0.4 *Group 4:* 2.7 ± 0.4	7 days

Bajkin et al., 2009 [17]	RCT	214	*Group A:* 62.1 ± 11.4 (31–79) *Group B:* 59.6 ± 11 (22–77)	Prosthetic valve replacement, atrial fibrillation, venous thromboembolic disease, ischemic heart disease, cerebrovascular accident, dilated cardiomyopathy, and hereditary thrombophilia	Liver or renal disease; pregnancy; being on drugs that alter the liver function or hemostasis; previous thromboembolic complications while on OAT; history of major bleed during dental extraction before starting OAT; history of heparin-induced thrombocytopenia	*Group A (n* *=* *109):* warfarin and acenocoumarol continued *Group B (n* *=* *105):* OAT stopped 3 to 4 days before the procedure with LMWH bridging	LMWH in group B	Dental extraction and no mucoperiosteal flap raised	*Group A:* resorbable collagen sponges, without sutures *Group B:* none, without sutures	*Group A:* INR < 4 *Group B:* INR < 1.5	*Group A:* 2.45 ± 0.54 *Group B:* 1.26 ± 0.11	1 month
Souto et al., 1996 [30]	RCT	92	*Initial study:* 59.7 ± 9.8 *Group 5:* 56.3 ± 9.4	Valvular heart disease (47 patients) or cardiac valve prosthesis (17 patients)	Previous thromboembolic complications while on OAT; history of major bleed during dental extraction before starting OAT; being on OAT for less than 3 months	*Groups 0, 1, and 2:* acenocoumarol's dose diminished before the procedure with calcium heparin use *Groups 3, 4, and 5:* OAT not changed and heparin not used. The antifibrinolytics used and postprocedural protocols varied between groups	None	Dental extractions	Epsilon-aminocaproic acid and tranexamic acid	*In native valves:* INR between 2 and 3 *In prosthetic valves:* INR between 2.5 and 4 *Only in group 5:* target INR was between 2 and 3 for an aortic prosthesis and from 2.5 to 3.5 for a mitral prosthesis or replacement of both valves	*Group 0:* 2.5 *Group 1:* 2.93 *Group 2:* 2.5 *Group 3:* 3.29 *Group 4:* 3.5 *Group 5:* 2.82	Unknown

Clemm et al., 2016 [33].	CCT	564	56 (18–92)	Atrial fibrillation, artificial heart valves, myocardial infarction, venous thromboembolism, pulmonary embolus, and cardiovascular prophylaxis	Acute or chronic sinusitis (in terms of planned implant placement in the upper jaw); drug or alcohol abuse and smoking; hematological diseases; metabolic, autoimmune, systemic, or immunological diseases; diseases that have an influence on blood coagulation or would negatively influence wound healing; chronic bone disease; untreated periodontitis; current steroid treatment; current chemotherapy; local radiation therapy; pregnancy	*Experimental (n* *=* *117):* being on one of the following: antiplatelets, VKAs, VKAs discontinued for 3 days with LMWH bridging, or NOACs (dabigatran, rivaroxaban, or apixaban). *Control (n* *=* *447):* no anticoagulation	LMWH in the experimental group	Implant and bone grafting surgeries	Sutures and electrocoagulation	Not mentioned in the study	*Bridging group:* 1.95 ± 0.47 *VKA group:* 2.62 ± 0.52	10 days
Cannon and Dharmar, 2003 [34]	CCT	70	*Experimental:* 62.4 (38–80) *Control:* 62.4 (36–78)	DVT, PE, TIAs, MI, arrhythmias, valvular disorders, prosthetic valve replacement, coronary artery bypass graft, stroke, and vascular thromboembolism	INR outside the therapeutic range of 2–4; history of liver disease; being on drugs affecting liver function	*Experimental (n* *=* *35):* warfarin continued *Control (n* *=* *35):* warfarin stopped 2 days prior to the procedure	None	Dental extractions, surgical removal, biopsies, closure of oroantral fistula, and mucoperiosteal flap sometimes raised	*Experimental:* none, except if removal of bone or damage to soft tissue *Control:* oxycellulose and sutures	*Experimental:* INR in the therapeutic range *Control:* INR < 2	*In all patients:* 3.4 (2.1–4) *Control:* 1.6 (1.4–1.9)	5 days

Devani et al., 1998 [35]	CCT	55	*Experimental:* 64.6 (30–82) *Control:* 61.3 (32–81)	DVT, PE, TIAs, MI, arrhythmias, valvular disorders, prosthetic valve replacement, coronary artery bypass graft, stroke, vascular thromboembolism, and dilated cardiomyopathy	INR outside the range of 2.0–4.0; history of liver disease; being on drugs affecting liver function and postoperative hemostasis	*Experimental (n* *=* *33):* warfarin continued *Control (n* *=* *32):* warfarin stopped 2 days prior to the procedure	None	Dental extractions and mucoperiosteal flap sometimes raised	Oxycellulose dressing and sutures	*Experimental:* INR in the therapeutic range *Control:* INR range of 1.5–2.1	*Experimental:* 2.7 (2–3.9) *Control:* 1.6 (1.2–2.1)	5 days

RCT = randomized controlled trial; CCT = controlled clinical trial; DVT = deep venous thrombosis; PE = pulmonary embolism; TIA = transient ischemic attack; MI = myocardial infarction; VKAs = vitamin K antagonists; NOACs = novel oral anticoagulants; OAT = oral anticoagulation therapy; LMWH = low-molecular-weight heparin.

**Table 2:** Outcomes of the studies.

Author and year of publication	Methods of assessing bleed	Bleeding outcome (*N* (%) or mean (range) or mean ± SD)	Need hospitalization for bleeding	Thromboembolic outcome (*N* (%))	Conclusions
Campbell et al., 2000 [31]	The difference of mass of sponges used in the procedure was then converted to volume The outcome was in “milliliters per unit of surgery”: a unit of surgery is a function of the surgical area involved and the risk of hemorrhage	*Experimental:* 1.4 mL/unit of surgery (0.1–4.5) *Control:* 2.2 (0.2–6.3) *Baseline:* 1.4 (0.6–2.1) No statistically significant difference	None	Not mentioned in the study	*OAT:* if INR is less than 3, warfarin can be continued in minor procedures, if there is an adequate surgical approach *Local hemostatic agents:* not needed when continuing warfarin

Evans et al., 2002 [27]	*Immediate bleeding:* if bleeding continues after 10 minutes of local pressure postprocedurally *Delayed bleeding:* if bleeding started > 10 minutes after the procedure Description of measures needed to interrupt the hemorrhage	*Experimental:* 15 (26%): 3 (5.2%) immediate and 12 (21%) delayed bleeding *Control:* 7 (14%) delayed bleeding No statistically significant difference (*P*=0.1)	Two patients in the anticoagulant group: one needed admission and the other presented to the ER without admission	Not mentioned in the study	*OAT:* if INR is in therapeutic range, warfarin can be continued in dental extractions done in a hospital setting with an increase in mild postprocedural hemorrhage *Number of teeth removed and risk of bleeding:* not associated

Erden et al., 2015 [32]	*Immediate bleeding:* this is estimated by the difference of mass of gauze swabs used in the procedure. The outcome in “milligrams” *Early bleeding:* this is estimated by the number of additional swabs needed during the first 48 hours	*Group A:* the amount of bleeding: 2194 ± 1418 mg; the median number of additional swabs used: 2.5; the median bleeding time: 50 *Group B:* the amount of bleeding: 2950 ± 1694 mg; the median number of additional swabs used: 3; the median bleeding time: 60 Greater immediate bleed in group B (*P* < 0.001) Greater early bleed in group B (*P* < 0.001) Greater bleeding time in group B (*P* < 0.001)	None	None	*OAT:* if INR is in therapeutic range, warfarin can be continued in dental extractions when patients have prosthetic valves *LMWH bridging:* this increases the risk of bleeding *Number of teeth removed and amount of bleeding:* positively correlated

Sacco et al., 2007 [28]	*Mild bleeding:* less than 10 minutes of duration *Moderate bleeding:* 10 to 20 minutes of duration *Severe bleeding:* this needs a new operation or a transfusion	*Group A:* 10 (15%) mild bleeding *Group B:* 6 (9.2%) mild bleeding No statistically significant difference (*P*=0.3)	None	None	*OAT:* if INR is in therapeutic range, warfarin can be continued in dental and alveolar procedures *Local hemostatic agents:* needed if warfarin is continued
Al-Mubarak et al., 2007 [29]	Bleeding assessed by a blinded examiner: *Bleeding is present*, if a fresh clot is eliminated without difficulty or if a discharge of blood is seen *Bleeding is absent*, if solid clot exists	*Group 1:* day 1: 12%, day 3: 4%, day 7: 0% *Group 2:* day 1: 21%, day 3: 3%, day 7: 0% *Group 3:* day 1: 17%, day 3: 3%, day 7: 4% *Group 4:* day 1: 29%, day 3: 5%, day 7: 0% No statistically significant difference, except groups 2 and 4 at day 3 (*P* < 0.05)	None	None	*OAT:* if INR <3, warfarin can be continued in dental extractions *Local hemostatic agents:* needed if warfarin is continued. Suturing should not always be performed *Number of teeth removed and risk of bleeding:* not associated *INR levels and postoperative bleeding:* positively correlated, but without any clinical significance

Bajkin et al., 2009 [17]	*Bleeding is noted*, when local pressure or further surgeries are needed *Immediate bleeding:* bleeding occurring until discharge *Late bleeding:* bleeding occurring after discharge	*Group A:* 8 (7.34%) had bleeding: 6 (75%) immediate and 4 (50%) late bleeding *Group B:* 5 (4.76%) had bleeding: 3 (60%) immediate and 3 (60%) late bleeding No statistically significant difference	None	None	*OAT:* if INR is in therapeutic range, VKAs can be continued in dental extractions *Local hemostatic agents:* needed if VKAs are continued. Suturing should not always be performed *LMWH bridging:* not needed for minor procedures *INR levels and postoperative bleeding:* no association Bleeding increases with local inflammation

Souto et al., 1996 [30]	*Mild bleeding:* hemorrhage ending alone or with mild pressure *Severe bleeding:* hemorrhage that requires more advanced methods to stop	*Group 0:* 85% mild, 15% severe bleeding *Group 1:* 50% mild, 50% severe bleeding *Group 2:* 64% mild, 36% severe bleeding *Group 3:* 83% mild, 17% severe bleeding *Group 4:* 69% mild, 31% severe bleeding *Group 5:* 96% mild, 4% severe bleeding There was no statistically significant difference between the groups when compared with group 0 So the risk of a major bleed is the same when reducing acenocoumarol with heparin use and when continuing the same dose with local antifibrinolytic use	Not mentioned in the study	Not mentioned in the study	*OAT:* if INR is in therapeutic range, acenocoumarol can be continued in dental extractions *Local hemostatic agents:* antifibrinolytic agent is needed, like tranexamic acid for two days, if acenocoumarol is continued *Heparin and reduced acenocoumarol given together* have multiple drawbacks *INR levels and postoperative bleeding* associated especially in groups that took reduced acenocoumarol with heparin *Number of teeth removed and risk of bleeding:* not associated
Clemm et al., 2016 [33]	*Immediate bleeding:* <24 h after the procedure *Delayed bleeding:* >24 h after the procedure *Low severity:* mild, controlled by local pressure *Moderate severity:* blood clots noticed, controlled by additional hemostatic methods *Severe:* bleeding artery noticed, controlled by more advanced methods	*Experimental:* on VKAs: low (6.7%); on VKAs bridged with LMWH: 1 (12.5%); on antiplatelets: 1 (1.6%); on NOACs: 0 (0%) *Control:* 3 (0.6%) There is a statistically significant difference between the VKA group and the control, where there is an increased risk of mild bleeding (*P*=0.038)	Two patients: one in the antiplatelet group and the other in the nonanticoagulated group	None	*OAT:* VKAs and NOACs can be continued during implant procedures, if the least invasive method is adopted, with an increase of mild postprocedural bleed in those on VKAs Implant surgery has a low bleeding risk regardless of the invasiveness of the procedure

Cannon and Dharmar, 2003	*Immediate bleeding:* up until 30 minutes after the procedure *Delayed bleeding:* >30 minutes Description of severity, time, and length	*Experimental:* 2 (5.7%) minor delayed bleeding *Control:* 3 (8.5%) minor delayed bleeding	None	None	*OAT:* if INR is in the therapeutic range, warfarin can be continued in minor procedures *Local hemostatic agents:* not needed

Devani et al., 1998 [35]	*Immediate bleeding:* up until 30 minutes after the procedure *Delayed bleeding:* >30 minutes Description of severity, time, and length	*Experimental:* 1 (3%) minor delayed bleeding *Control:* 1 (3.1%) minor delayed bleeding	None	None	*OAT:* if INR is in therapeutic range, warfarin can be continued in dental extractions if no other medications are taken that affect the liver or hemostasis *Local hemostatic agents:* needed

VKAs = vitamin K antagonists; NOACs = novel oral anticoagulants; OAT = oral anticoagulation therapy; LMWH = low-molecular-weight heparin.

**Table 3:** Recommendations for specific dental procedures based on corresponding RCTs, CCTs, and/or expert opinion.

Dental procedure	Risk of bleeding	Number of RCTs and CCTs dealing with the procedure	Recommendation for VKA and NOAC use preprocedurally
Surgical teeth extraction	Low	RCTs: 4^*∗*^ [17, 27, 29, 30] + 1^*∗∗*^ [28] CCTs: 2^*∗*^ [32, 35] + 2^*∗∗*^ [31, 34]	VKAs should be continued if INR is in therapeutic range [17, 27, 28, 30, 32, 34, 35] or <3 [29, 31] Local hemostatic agents were judged essential in most studies [17, 28–30, 35] NOACs: no RCTs or CCTs available yet *Expert opinion*: continue NOACs with caution with local hemostatic agents

Implant surgery	Low [33]	RCTs: 1^*∗∗*^ [28] CCTs: 1^*∗*^ [33]	VKAs: continue anticoagulation if INR is in therapeutic range [28, 33] with use of local hemostatic agents [28] Continue anticoagulation with NOACs [33]

Excision of cystic formations	Low (*Expert opinion*)	RCTs: 1^*∗∗*^ [28] CCTs: 0	VKAs must be continued if INR is in therapeutic range, with the use of local hemostatic agents [28] NOACs: no RCTs or CCTs available yet *Expert opinion*: continue NOACs with caution with local hemostatic agents

Biopsy	High [36]	RCTs: 0 CCTs: 1^*∗∗*^ [34]	VKAs must be continued if INR is in the therapeutic range. No local hemostatic agents are needed [34] NOACs: no RCTs or CCTs available yet *Expert opinion*: continue NOACs with caution with local hemostatic agents

Alveoloplasty	Moderate (*Expert opinion*)	RCTs: 0 CCTs: 1^*∗∗*^ [31]	VKAs must be continued if INR is less than 3. No local hemostatic agents are needed [31] NOACs: no RCTs or CCTs available yet *Expert opinion*: NOACs must be continued along with local hemostatic agents

Frenectomy	Moderate (*Expert opinion*)	RCTs: 0 CCTs: 1^*∗∗*^ [31]	VKAs must be continued if INR is less than 3. No local hemostatic agents are needed [31] NOACs: no RCTs or CCTs available yet *Expert opinion*: NOACs must be continued along with the use of local hemostatic agents

Periodontal surgery	High if raising a flap is needed [36]	RCTs: 0 CCTs: 0	*Expert opinion*: continue oral anticoagulation as scheduled if INR is within the therapeutic range (if VKAs), with the use of local hemostatic agents

Endodontic microsurgery (apicectomy)	High [37]	RCTs: 0 CCTs: 0	*Expert opinion*: continue anticoagulation with caution with local hemostatic measures

^*∗*^The corresponding RCTs or CCTs deal only with the unique procedure cited above. ^*∗∗*^The corresponding RCTs or CCTs deal with multiple procedures, among which one has been cited. RCT  =  randomized controlled trial; CCT  =  controlled clinical trial; VKAs = vitamin K antagonists; NOACs = novel oral anticoagulants; OAT = oral anticoagulation therapy.
